# Nap patterns of children in kindergartens and childcare transit facility: a study in northern Peninsular Malaysia

**DOI:** 10.5935/1984-0063.20220011

**Published:** 2022

**Authors:** Mai Fujii, Masayuki Hayashi, Cheong Lieng Teng

**Affiliations:** 1 Doshisha University, Center for Baby Science - Kizugawa - Kyoto - Japan.; 2 Khon Kaen University, Faculty of Public Health - Khon Kaen - Muang - Thailand.; 3 International Medical University, Department of Family Medicine - Seremban - Negeri Sembilan - Malaysia.

**Keywords:** Sleep, Child Behavior, Malaysia, Child Care, Child, Preschool, Asia, Southeastern

## Abstract

**Objective:**

The purpose of this study was to compare the duration of sleep and nap patterns between children in private kindergartens and those in a *tabika*-transit facility, in Malaysia.

**Methods:**

The sleep duration of children aged 3-6, who attended either a kindergarten or a childcare transit facility (*tabika*-transit in Malay) was investigated. Observational sleeping records were maintained for 24 hours, over 14 consecutive days. A self-administered questionnaire for caregivers investigated the children’s lifestyles. Naps were optional at kindergartens but mandatory at the *tabika*-transit.

**Results:**

Of 35 participating children, data from 33 were analyzed. All respondents belonged to the Malay ethnicity, with 16 boys (48.5%) and 17 girls (51.5%). The average age of the children was 5.4 years; 11 of them were from kindergartens and 22 from a *tabika*-transit. The children slept longer and woke up later on weekends than on weekdays. There was a significant difference in the naptaking rate between the two groups; it was 100% in the *tabika*-transit, and 30% in the kindergartens during weekdays. However, on weekends, 19 of 22 *tabika*- transit children did not nap on any of the days (86.4%). The kindergarten group’s naps showed no outstanding differences between weekdays and weekends. Concerning the bedtime and wake-up times, no differences were found between the two groups.

**Discussion:**

During weekdays, all children in the *tabika*-transit took naps, whereas one-third of them did in the kindergartens. Larger study is needed to assess how this mandatory napping style affects children’s lifestyle and development.

## INTRODUCTION

Acquiring adequate sleeping rhythms and patterns is crucial to the overall development of a child. Research has shown that a child’s sleeping pattern undergoes many changes during the first five years of their life ^1^. In early childhood, sleeping is considered the brain’s primary activity, and Dahl ^2^, alongside other researchers, reported that health risks are more likely to occur in later years if sleeping rhythms and patterns are inadequate in the early stages of life ^3^.

A feature of sleep that is relatively unique to early childhood is nap-taking. For preschool-aged children, nap-taking is an essential part of their total sleeping time and supplements night-time sleep. As children grow up, nap time and frequency is reduced ^4^; by the age of three years or above, the proportion of children who skip naps is over 80% ^5^. Sleeping hours and patterns also differ according to family environment, culture, or ethnicity ^6,7,8^. A study comparing 3–6-year-old children in Asian and Caucasian countries reported that children stopped napping at 5 years in Caucasian countries, but continued doing so beyond this age in Asian countries ^9^. This study also reported that cross-cultural temporal differences were found in sleep patterns, but not in the total duration of sleep that children had over 24 hours.

Social environment is another factor that affects children’s sleep rhythms and patterns. Nowadays, many pre-school children attend childcare facilities where they spend most of their daytime. However, there have been few reports on nap-taking patterns of the children attending these facilities. A study of nocturnal sleep and daytime nap behaviours in preschool-age children attending childcare highlighted that understanding children’s sleep patterns, how they interact with daily stresses, and their biobehavioural responses is important ^10^. Some existing studies have highlighted the importance of proper balance or rhythms in the schedules of day care facilities ^11,12^.

Research has shown that nap-taking hours should decrease as children grow; however, there are facilities that continue to offer unnecessary naps to growing children ^13^. Fukuda indicated that scheduling a group naptime for all children in a nursery could result in psychological distress, because children at the age of 4 or 5 no longer biologically require a nap 5. A study on nap programs in Japanese kindergartens and nurseries found that obligatory nap programs were being conducted in some nurseries despite the lack of biological requirement. These researchers recommended that unnecessary nap programs in nurseries for older preschool children be terminated ^14^.

One of the reasons underlying the issue of unnecessary naps in Japanese childcare facilities is the previous existence of national guidelines for the management of pre-school facilities such as kindergartens and nurseries. Kindergartens and nurseries are managed by different national laws and curriculums. The primary purpose of kindergarten is early education and the target age is 4 to 6, and therefore, nap-taking is not indicated in the management guidelines. Most kindergartens, in accordance with their guidelines, set nap-taking as optional. However, the guidelines were initially developed for nurseries, which cater to a younger age group (i.e., below 3 years of age) than the children in kindergartens. Therefore, “nap-taking” was included in the Japanese guidelines for nursery management until 2008; however, it was abolished in 2009, because nurseries now cover a wider range of children and it is evident that not all children require naps. Nonetheless, such a longstanding custom cannot be easily done away with. Many nurseries in Japan have continued implementing obligatory nap-taking programs.

Japan has provided other countries with official development assistance (ODA) in the area of early child education. Japan had been a top donor for ODA ^15^ to Malaysia and provided technical assistance for early child education since the early 2000s ^16^. Therefore, this study aimed to assess the recent trend on pre-school children’s nap-taking patterns in Malaysia. While there are existing studies on nap programs, literature on nap-taking in different childcare facilities is scarce. Therefore, this study focused on children in kindergartens and nurseries to compare naptime practices in different childcare facilities. In Malaysia, the composition of children under 5 years was 8% (2.6 million) of the total population in 2018 ^17^. The majority of children use childcare service facilities, and the enrolment ratio of pre-primary facilities reached 92% for boys and 96% for girls ^18^. Services for preschool children in Malaysia are mainly divided into two: nursery (*taska* in Malay) for children under 3 years old, and kindergarten (*tadika* or *tabika* in Malay) for children older than 3. This study focused on the children who attend kindergarten. The laws upon which kindergartens and nurseries base their management differ according to the facility type. Kindergartens tend to place more weight on providing pre-school education ^19^, whereas nurseries focus more on providing care for very young children.

A unique feature of Malaysian public kindergartens is that they finish their services by noon ^20^, and therefore, some of the children use another childcare transit facility (known as *tabika-*transit in Malay) in the afternoon, if their parents are working ^21^. Most *tabika-*transit facilities are not recognized by national authorities. Their staff consists of carers who take care of children by applying a nursery care model targeted at young children aged 0–3. Therefore, private kindergartens which stay open all day often focus on education even in the afternoon, with an optional nap schedule, whereas *tabika-*transits often schedule mandatory naps even for older preschool children aged 3 to 6. Hence, we examined and assessed the differences in nap schedules between two groups of children: one in a private kindergarten and the other in a *tabika-*transit facility.

## MATERIAL AND METHODS

The survey data used here was collected from December 2019 to February 2020 as a pilot study for a larger survey. However, due to COVID-19, the subsequent survey has been postponed until circumstances allow the implementation of a large-scale survey. In this cross-sectional study, data was obtained from childcare facilities in northern Malaysia. Four facilities agreed to participate in the survey; three were private kindergartens offering all-day programs, and one was a *tabika*-transit. From these, 35 children and their guardians agreed to participate. Of the eligible participants, the ones who withdrew in the middle of the survey and the ones who provided incomplete information were excluded. Therefore, the final number of responses included in the analysis was 33.

**Figure 1:**
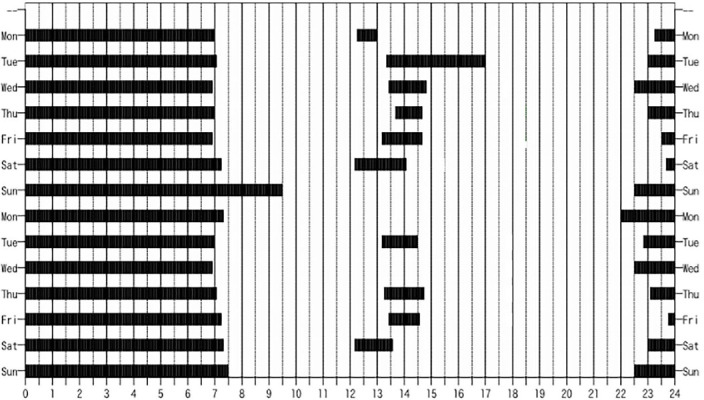
Examples of a sleep log.

The survey included a one-time, self-administered questionnaire and an observational sleep-record of 14 consecutive days for each child. Both the caregivers and the staff in these facilities documented descriptive 24- hour records of the children’s sleeping patterns for two consecutive weeks, using the sleep log developed by Miike (22, 23). In the sleep log, information on bedtimes and wake up-times, amount of time spent asleep at night and during naps, waking periods during sleep-time (midwake time), and the duration of all sleeping events were recorded using a hand-drawn line or mark in the sleep log. An example of this sleep log is shown in Figure 1.

Information on self-waking, defecation upon waking, and whether the child ate breakfast each morning was also recorded on each of the 14 days. The staff in the facilities kept the naptime records and the children’s caregivers kept records of all other sleep times at home, during the night, and on weekends. The children’s caregivers at home also responded to a self-administered questionnaire to assess the children’s respective lifestyles. In addition to the sociodemographic characteristics of the participants, questions regarding children’s media use before sleeping at night and whether they went out after 20:00 were also included. To measure children’s media use habits before going to sleep at night, a question regarding whether parents let their child go to sleep by watching television, DVDs (digital video disc), or YouTube videos was included. Parents were also asked whether they went out with their child after 20:00 (e.g., to a convenience store). Specifics regarding the terminology utilized in this study are shown below.

### Kindergarten and tabika-transit

All three private kindergartens in this study provided all-day programs. The *tabika*-transit only offered an afternoon program and accepted children after their public kindergarten finished at noon, which is the usual practice in Malaysia. There was no nap program in the morning at the kindergartens. All-day kindergartens in this study offered educational programs in the morning and optional nap programs in the afternoon. Whereas, the *tabika-*transits in this study scheduled mandatory naps. In these facilities, naptime started under staff supervision at around 14:00.

### Weekdays and weekends

In Malaysia, weekdays and weekend differ by states. For example, Sunday is a non-working day in some states and a normal working day in other states. In this study, weekdays are defined as the five days of a week that children go to kindergarten and/or a *tabika-*transit. Weekends are defined as the remaining two days of the week, considered to be holidays (when children do not attend these facilities).

### Duration of sleep

The overall duration of sleep was calculated as the sum of night-time sleep and naptime sleep, if a nap was taken. All sleep durations were calculated by deducting wake-up times from bedtimes. If a mid-wake was recorded, the duration of this wake time was deducted. The event of one day is expressed by using the unit of person-day; one person-day consists of night-time sleep and a naptime sleep, if taken. The frequency of naps and wake times per day was also counted.

### Statistical analysis

A comparison analysis of weekday sleep vs. weekend sleep was conducted on children attending kindergartens and children attending a *tabika*-transit by using a univariate analysis. For the comparison of the two groups, Wilcoxon signed- rank test (Man-Whitney U test) was used. All sleeping events were first consolidated and overall sleep patterns were assessed by calculating the mean of bedtimes and wake-up times, duration of sleep per day, and frequency of naps per day. The relationship between sleep patterns and lifestyle factors, such as media use before sleeping or going out at night, were also assessed. The level of significance was two-tailed (0.05 significance level). All analyses were conducted using IBM SPSS Statistical Software version 26 (SPSS, Inc, Chicago, IL, USA).

### Ethical clearance

We conducted a briefing session with all the participants and obtained informed consent from the staff at the facilities and from the guardians of the children. This study was approved by the Doshisha University Research Ethics Committee (approval number: 19074, 2020). Due to the COVID-19 pandemic, the main survey has been postponed until circumstances allow for it to be conducted.

## RESULTS

Out of 35 children, one parental consent was withdrawn, and one caregiver did not complete the questionnaire; therefore, 33 questionnaires were analyzed. Table 1 shows the basic characteristics of the participants. From the 33 responses, we learned that the children’s mean age was 5.4 years ± 1.0 (SD) and that all children were of Malay ethnicity. There were 16 boys (48.5%) and 17 girls (51.5%) among the children, with no significant differences based on gender. One-third of the children attended kindergartens where naps were optional, and the remaining two-thirds attended a *tabika*-transit where naps were mandatory. In both facilities, group napping patterns were adopted, with two separate rooms prepared for girls and boys. A small percentage (24.2%) of caregivers responded that they let their children use electronic media, such as YouTube videos or DVDs, before sleeping. A small group (21.2%) indicated that they went out with their children after 20:00. No association was found between these lifestyle factors and children’s sleep patterns, such as bedtimes and wake-up times or duration of sleep.

**Table 1:** Socio-demographic characteristics of the participants (N=33).

Variables			N	%
Age of children (mean ± SD, year		5.4 ± 1.0	33	-
	3 years		2	6.1
	4 years		6	18.2
	5 years		13	39.4
	6 years		12	36.4
Sex of children	Male		16	48.5
	Female		17	51.5
Types of facilities that a child goes during weekdays	Kindergarten		11	33.3
	Tabikatransit		22	66.7
Let a child sleep by let him/her watch TV, DVD, or YouTube for nighttime sleep	No, relatively No		25	75.8
	Yes, relatively Yes		8	24.2
Going out with a child after 20:00 (example: to a convenience store	No, relatively No		26	78.8
	Yes, relatively Yes		7	21.2
Age of mother (mean ± SD, yea		34.6 ± 4.2	30	-
Mother’s highest level of education (N=30)	Junior high school		4	13.3
	High school or junior college		9	20.0
	University or high		17	56.7
Age of father (mean ± SD, year)		37.1 ± 6.0	29	-
Father’s highest level of education (N=29)	Junior high school		2	6.9
	High school or junior college		12	41.4
	University or higher		15	51.7

The 14 observation days included 10 weekdays and 4 weekend days. All children slept each night; however, there were children who did not take any naps during the observation period. Table 2 presents the bedtimes and wake-up times of the children, grouped by weekdays and weekends. The average wake-up times of night-time sleep were 7:27 ± 1:06 (SD) on weekdays, and 7:52 ± 1:38 (SD) on weekends, showing a significant difference. Six children did not take any naps during the survey period. For the 27 children who took naps on at least one day during the survey period, the average bedtime and wake-up times were 13:57 ± 0:31 (SD) and 16:52 ± 0:50 (SD), respectively. There were significant differences between wake-up times and duration of night-time sleep during weekdays and weekends. The average duration of night-time sleep of all children was 9 hours 32 minutes ± 1 hour 25 minutes (SD) on weekdays, and 10 hours 4 minutes ± 1 hour 44 minutes (SD) on weekends, showing a significant difference.

**Table 2:** Bed- and wake-up time by weekdays and weekends (N=33).

Variables	All (h:mm) Mean ± SD	Weekdays (h:mm) Mean ± SD	Weekends (h:mm) Mean ± SD	P Value
Bedtime (night)	21:47 ± 0:49	21:49 ± 0:51	21:44 ± 0:56	NS
Wake-up time (night)	7:35 ± 1:11	7:27 ± 1:06	7:52 ± 1:38	0.022
Bedtime (nap)	13:57 ± 0:31*1	13:59 ± 0:35*1	13:36 ± 1:01*2	NS
Wake-up time (nap)	16:52 ± 0:50*1	16:52 ± 0:57*1	15:48 ± 0:55*2	NS
Duration of sleep (all)*^3^	11:26 ± 1:35	11:50 ± 1:45	10:29 ± 1:47	0.001
Duration of sleep (night)*^3^	9:39 ± 1:27	9:32 ± 1:25	10:04 ± 1:44	0.005
Duration of sleep (nap)*^3^	2:54 ± 0:42*1	2:55 ± 0:41*1	2:11 ± 1:11*2	NS

*1: N=27 (six children who did not take any nap during the weekdays)

*2: N=6 (27 children who did not take any nap during the weekend) Mid-wake time was excluded from duration of sl.

**Figure 2:**
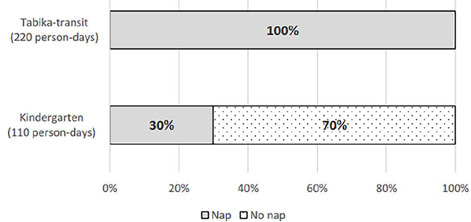
Nap taking rate on weekdays by facility.

**Figure 3:**
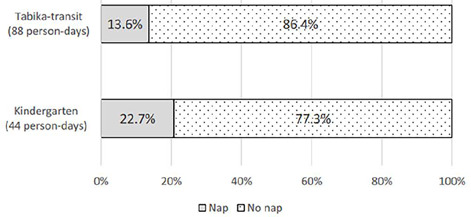
Nap taking rate on weekend (at home).

There were 14 days of records for each child; thus, 462 person-day records were obtained from 33 children. All naps were scheduled in the afternoons and, if taken, occurred a maximum of once a day. Figure 2 presents the nap-taking rate on weekdays by facility groups and Figure 3 presents the rate for weekends. During weekdays, all children in the *tabika*-transit took a nap each day, whereas only 30% of children in kindergartens took naps each day. In the kindergarten group, 6 of the 11 children did not take naps on any weekday. On weekends, 19 of 22 *tabika*-transit children did not nap on any of the days (86.4%). In the kindergarten group, 8 children (72.7%) did not take any naps on weekend days, 2 (18.2%) took one nap in four weekend days, and 3 (36.4%) took naps for all four weekend days. On weekends, the proportion of non-napping children was 77.3% for kindergarteners and 86.4% for *tabika*-transit children, without a significant difference.

The duration of sleep on weekdays and weekends by facility type is shown in Table 3. The average duration of total sleep was significantly different; 10 hours 37 minutes ± 1 hour 31 minutes (SD) for kindergarten children, and 12 hours 26 minutes ± 1 hour 33 minutes (SD) for *tabika*-transit children for weekdays; 11 hours 02 minutes ± 1 hour 33 minutes (SD) for kindergarten children, and 10 hours 12 minutes ± 1 hour 52 minutes (SD) for *tabika*-transit children for weekends. There was no significant difference observed in the duration of night-time sleep between the facility groups. With respect to nap-taking, 5 out of 11 children did not take nap at all in the kindergarten group, whereas all 22 children took a nap in the *tabika*-transit group. The average duration of a nap in Table 3 was calculated only on days when the children slept, and there were no significant differences between the groups for the weekdays’ naps; however, nap duration over the weekends was significantly different.

**Table 3:** Duration of sleep on weekdays and weekends by facility type.

	Variables	Kindergarten (N=11) Mean ± SD (h)	Tabika-transit (N=22) Mean ± SD (h)	P value
Weekdays	All	10:37 ± 1:31	12:26 ± 1:33	0.01
	Night	9:50 ± 1:33	9:23 ± 1:22	NS
	Day (nap)	2:44 ± 0:29*4	2:58 ± 0:43	NS
Weekends	All	11:02 ± 1:33	10:12 ± 1:52	0.04
	Night	10:23 ± 1:22	9:55 ± 1:54	NS
	Day (nap)	2:56 ± 1:17*5	1:26 ± 0:24*6	0.05

*4: N=5 (6 children who did not take nap are not included)

*5: N=3 (8 children who did not take nap are not included)

*6: N=3 (19 children who did not take nap are not included) Mid-wake time was excluded from duration of sleep.

When comparing average nap time including the children who don’t sleep on weekends, the nap duration become one-thirds than that of the weekdays, however SD is very large.

Table 4 presents the bedtimes and wake-up times for night-time and naptime sleep. Children in both the kindergartens and *tabika*-transit went to sleep before 22:00 and woke up between 7:00 to 8:00. No significant difference was found for bedtime and wake-up time between the two groups. On comparing the children who took naps, there was no difference in bedtime and wakeup times for naps in both groups.

**Table 4:** Comparison of bed- and wake-up times between kindergarten and Tabika-transit.

Variables	Bedtime/wake-up time	Kindergarten (N=11) Mean ± SD (h)	Tabika-Transit (N=22) Mean ± SD (h)	P value
All days	Bedtime (night)	21:47 ± 1:01	21:46 ± 0:43	NS
	Wake-up time (night)	7:52 ± 0:49	7:26 ± 1:19	NS
	Bedtime (nap)	13:54 ± 1:04*7	13:58 ± 0:20	NS
	Wake-up time (nap)	16:35 ± 0:43*7	16:56 ± 0:52	NS
Weekdays	Bedtime (night)	21:52 ± 1:05	21:47 ± 0:44	NS
	Wake-up time (night)	7:45 ± 0:51	7:18 ± 1:12	NS
	Bedtime (nap)	14:05 ± 1:17*7	13:58 ± 0:20	NS
	Wake-up time (nap)	16:49 ± 1:15*7	16:53 ± 0:54	NS
Weekends	Bedtime (night)	21:36 ± 1:14	21:46 ± 0:46	NS
	Wake-up time (night)	8:07 ± 1:06	7:45 ± 1:52	NS
	Bedtime (nap)	13:33 ± 1:30*8	13:44 ± 0:30*9	NS
	Wake-up time (nap)	16:30 ± 0:47*8	15:49 ± 1:27*9	NS

*7: N=5 (6 children who did not take nap are not included)

*8: N=3 (8 children who did not take nap are not included)

*9: N=3 (19 children who did not take nap are not inclued).

## DISCUSSION

There were no significant differences in bedtimes and wake-up times between the kindergarteners and *tabika*-transit children on weekdays or weekends. Some studies have reported that nursery school children with obligatory naps have later bedtimes, but our results did not support this finding ^14,24^. A study of Iranian preschool children reported that earlier sleepers had a longer night-sleep duration ^25^. In our study, no evidence was found that *tabika*-transit children who took an average of 3-hour naps slept later or got shorter sleep at night compared to kindergarteners. A study of 121 Malaysian children aged 3–6 found that their average bedtime and wake-up time was 10:17 and 7:63, respectively ^9^, which is a later bedtime than that revealed by our research. In another study of children aged 1.5 in Japan, nap wake-up time was reported to be at approximately 15:13 ^24^. Our results showed a much later wake-up time for naps, which might be due to cultural and societal differences. Although there was a difference in the wake-up time from night-time sleep between weekdays and weekends, both facility groups comparison showed similar tendencies. While previous studies have suggested that inappropriate nap patterns and late naps may induce late bedtimes ^24^, our study found no such evidence. However, another study has indicated that the interval between naptime and night-time sleep becomes longer as children grow ^26^. Thus, further assessment of this factor should be carried out in future studies.

The National Sleep Foundation which is convened a multidisciplinary expert panel from leading stakeholder organizations and the American Academy of Sleep Medicine indicated that pre-schoolers (3–5 years old) should obtain 10–13 hours of sleep ^27,28^. The results obtained in this study were within this range, at 11.26 ± 1.35 hours (SD). A study of Malaysian pre-schoolers reported that the average sleep-time was 10.83 hours ^9^. In comparison with weekdays, children had longer nighttime sleep and woke up later on weekends. In a study on weekend catch-up sleep, a relationship between longer weekend sleep and obesity was reported for elementary school children ^29^. Longer weekend sleeping habits might start from the preschool age or earlier, and therefore, further research is needed to investigate sleeping patterns in younger children. The average nap duration in this study was almost 3 hours, which is longer than those found in previous studies. A study conducted using actigraphy on 1.5-year-old children reported that the average duration of naps was 1.9 hours ^24^. In the study of Malaysian children, the average duration was 1.73 hours ^9^. After measuring sleeping hours by means of a digital device, that is, actigraphy, Tikotzky reported that the mean nap time for children aged 5.5 years was 12.7 minutes ^30^. The average nap time this study was 2 hours 54 minutes ± 42 minutes (SD), which is much longer. A previous study reported that the total duration of sleep does not differ even if the timing of the sleep rhythm has variations ^9^; our results did not support this. Since the records in our study are observational, cases wherein children may be pretending to sleep to follow the nap schedule were also counted as sleep. Existing studies indicate that there is a negative correlation between the duration of night and daytime sleep among children aged 2.5 to 4.5 years ^31^. While many children in kindergartens do not take naps, all children in *tabika*-transits do. A study relating to mandatory nap time and group napping patterns indicated that exposure to longer mandatory nap times in childcare may increase the incidence of napping ^32^. However, in relation to naps and night-time sleep, our results did not show a significant difference.

A previous study reported that most children aged 6 years do not take any naps in their home environment ^5^. In this study, the results collected from the weekend records also support this finding; when at home on weekends, 27 out of the 33 children did not take any naps. A past study conducted by Weissbluth reported that the peak age at which children stop napping is 4 years ^33^. However, our study showed that all children over 4 years old took naps on all weekdays in the *tabika*-transit. With respect to nap times per day, our results showed that it was 0.6 times with an average age of 5.4. Previous studies have shown variations regarding this. One study reported that the average number of naps taken per day was 0.88 times in 3-year-old children, 0.72 times in 4-year-olds, 0.58 times in 5-year-olds, and 0.43 times in 6-year-olds ^33^.

### Limitations of the study

Due to the small sample size, the results of this study are preliminary. The sleep measurement was conducted by the childcare facility staff for naps and by the children’s guardians for the rest of the sleep on weekdays; this might have led to variations in individual manual methodology. Therefore, this study might not have the same accuracy level as that shown by electronic devices such as actigraph.

## CONCLUSION

Only one-third of the children in kindergarten (optional nap) took naps, compared to all the children in the *tabika*-transit during weekdays. Thus, in this study, a high nap-taking rate was found in the *tabika*-transit. However, proper interpretation of mandatory naps requires further, large-scale research, to assess long-term health implications or educational benefits or harm for children.
